# New Algorithms for Autonomous Inertial Navigation Systems Correction with Precession Angle Sensors in Aircrafts

**DOI:** 10.3390/s19225016

**Published:** 2019-11-17

**Authors:** Danhe Chen, Konstantin Neusypin, Maria Selezneva, Zhongcheng Mu

**Affiliations:** 1 Nanjing University of Science and Technology, Nanjing 210094, China; 2 Moscow Bauman State Technical University, Moscow 105005, Russia; neysipin@mail.ru (K.N.); m.s.selezneva@mail.ru (M.S.); 3 School of Aeronautics and Astronautics, Shanghai Jiao Tong University, Shanghai 200240, China

**Keywords:** aircraft, autonomous inertial navigation system, precession angle sensor, nonlinear Kalman filter

## Abstract

This paper presents new algorithmic methods for accuracy improvement of autonomous inertial navigation systems of aircrafts. Firstly, an inertial navigation system platform and its nonlinear error model are considered, and the correction schemes are presented for autonomous inertial navigation systems using internal information. Next, a correction algorithm is proposed based on signals from precession angle sensors. A vector of reduced measurements for the estimation algorithm is formulated using the information about the angles of precession. Finally, the accuracy of the developed correction algorithms for autonomous inertial navigation systems of aircrafts is studied. Numerical solutions for the correction algorithm are presented by the adaptive Kalman filter for the measurement data from the sensors. Real data of navigation system Ts-060K are obtained in laboratory experiments, which validates the proposed algorithms.

## 1. Introduction

In the development of control systems for emerging dynamic objects, various navigation systems have been considered [1,2]. In particular, for aircrafts (AC) the system performance is largely determined by assurance of the quality of both measuring systems and obtained signals for flight control. For example, inertial navigation systems (INS), etc. usually are used in atmospheric AC measuring systems among various gyroscopic navigation systems [3,4]. At present, all modern INS have different designs and the free platform INS have obtained increasing popularity. However, in practice, INS with a gyro-stabilized platform (GSP) are more reliable and better developed, which makes them preferable for successful application on AC [5]. Furthermore, the accuracy of an INS can be improved by acquiring additional information. Usually, this means information external to the INS from various sensors and systems. 

The satellite navigation systems are well known for being highly accurate and relatively low cost. However, they are subject to interference and classified as autonomous systems [6,7]. Autonomous systems such as air signal systems, Doppler velocity, drift meters and others, all have high reliability and autonomy, but at the same time with lower accuracy. Astro-inertial systems are considered the most accurate among other navigation systems [8,9], while in another aspect it is recognized that these astro-systems have a lower noise immunity. During the operation time of autonomous INS with sufficiently long intervals, errors can reach unacceptably large values [10]. It is necessary to compensate for the errors of an autonomous INS applying the internal connections of the system. 

Recently, most researches have focused on the correction algorithm through Kalman filter (KF) [11,12,13]. Algorithms for the compensation of autonomous INS errors by means of forming correction units and internal connections of the system have been widely investigated, applied, and developed in detail. For instance, the compensation of INS errors through KF without external measurements is known for the horizontal movement of an INS-carrying object with a constant velocity [14]. In addition, the error equations of an autonomous INS are used as equations of the object in Kalman filter, and the signals from accelerometers under conditions of the aircraft motion at a constant velocity are taken as measurements. In practical applications, an aircraft generally performs a complex accelerated motion, which leads to the verdict that the mentioned method is rarely used to compensate for autonomous INS errors. In [15] Chingiz developed a modified optimum Kalman Filter with INS error compensation for flight dynamics estimation of an aircraft. Wei et al. [16] proposed an autonomous positioning method independent of satellites based on principle of star sensor and inclinometer, and established the measurement model of the system. While the errors compensation during the flight of objects remains as a major problem in order to improve the accuracy of autonomous systems.

The most complete algorithmic compensation of INS errors was carried out by methods of integration and subsequent processing of information through estimation algorithms [17,18,19,20]. Usually, the correction of the navigation system is performed with external sources of information [21,22]. Reference [23] developed a low-cost INS provided by the respective sensors and mathematical modeling of the errors for vehicular navigation, where the evaluation is processed still by GPS aiding. However, in the case of correction from external measuring systems, the INS may lose such valuable quality as an autonomous INS. In the present work, only autonomous INS and correction methods for AC due to internal information are considered, without using other measuring systems. New methods for correction of autonomous INS, based on an original approach of formation in a measurement signal using information from precession angle sensors, have been developed. Meanwhile, a semi-physical experiment with a serial system of the second class of accuracy, Ts060K, has been conducted and a verification of efficiency of the developed algorithms is performed based on the simulation and experiment results. The method for compensating dynamic errors of an autonomous INS [24] involves generation of correction signals to the moment sensors and the first integrators using information from precession angle sensors. In this case, the compensation is performed for the dynamic components of errors, and the correction signals are formed on the basis of solving equations of INS errors in the first approximation. It is reasonable to compensate these errors in INS equipped with such similar algorithms due to the residual errors, which are caused by various perturbing factors.

The structure of this paper is as follows. New errors models of autonomous navigation systems and the method of measurement generation based on signals from precession angle sensors are described in Section 2. Correction algorithm of an autonomous INS by Kalman filter is presented in Section 3. Section 4 is concerned with the correction of an autonomous INS using the reduced measurements. A method of forming correction signals using non-linear error equations in INS is developed. Section 5 discusses the simulation results of autonomous INS correction algorithms by signals from precession angle sensors. An analysis of simulation results is also given for autonomous INS correction algorithms. Finally, conclusions are presented in Section 6.

## 2. Generation of Errors and Measurements for Autonomous INS

### 2.1. Errors of Autonomous Navigation Systems

In this section, non-linear models of INS errors at optimal level are presented in detail. Generally, the errors models can fairly precisely reflect the main features of the changes in INS errors and they can easily be implemented in a special calculator or on-board computer of aircraft.

Accordingly, the non-linear error equations of autonomous INS are expressed as follows [25,26]:

(1) $$\begin{array}{l} \delta {\dot{v}}_{E}=-f_{E}-v_{up}(\frac{\delta v_{E}}{R}-u\sin\varphi \delta \varphi )+v_{N}(\frac{\delta v_{E}}{R}tg\varphi +u\cos\varphi \delta \varphi +\frac{v_{E}}{R}\sec^{2}\varphi \delta \varphi -\delta v_{up}(2u\cos\varphi +\frac{v_{E}}{R})+\\ +\delta v_{N}(2u\sin\varphi +\frac{v_{E}}{R}tg\varphi )-f_{up}\cos\Phi_{E}\sin\Phi_{N}+f_{E}\cos\Phi_{N}\cos\Phi_{up}+f_{N}(\cos\Phi_{N}\sin\Phi_{up}+\\ +\sin\Phi_{E}\sin\Phi_{N}\cos\Phi_{up})-f_{E}\sin\Phi_{E}\sin\Phi_{N}\sin\Phi_{up}+f_{E}\mu_{E}+B_{E};\\ \delta {\dot{v}}_{N}=-f_{N}-v_{up}\frac{\delta v_{N}}{R}-v_{E}(\frac{\delta v_{E}}{R}tg\varphi +u\cos\varphi \delta \varphi +\frac{v_{E}}{R}\sec^{2}\varphi \delta \varphi )-\delta v_{up}\frac{v_{N}}{R}-\delta v_{E}(2u\sin\varphi +\frac{v_{E}}{R}tg\varphi )+\\ +f_{N}\cos\Phi_{E}\cos\Phi_{up}-f_{E}\cos\Phi_{E}\sin\Phi_{up}+f_{up}\sin\Phi_{E}+f_{N}\mu_{N}+B_{N};\\ {\dot{\Phi }}_{E}=(-\frac{v_{E}}{R}-\frac{\delta v_{N}}{R}+\omega_{E}^{dr})\cos\Phi_{N}+\frac{v_{N}}{R}\cos\Phi_{up}-(\frac{v_{E}}{R}+u\cos\varphi )\sin\Phi_{up}+(\frac{v_{E}}{R}tg\varphi +u\sin\varphi +\\ +\frac{\delta v_{E}}{R}tg\varphi +u\cos\varphi \delta \varphi +\frac{v_{E}}{R}\sec^{2}\varphi \delta \varphi +\omega_{up}^{dr})\sin\Phi_{N};\\ {\dot{\Phi }}_{N}=\frac{v_{E}}{R}+u\cos\varphi +\frac{\delta v_{E}}{R}-u\sin\varphi \delta \varphi +\omega_{up}^{dr}-\frac{v_{N}\sin\Phi_{up}}{R\cos\Phi_{E}}-(\frac{v_{E}}{R}+u\cos\varphi )\frac{\cos\Phi_{up}}{\cos\Phi_{E}}+\\ +(-\frac{v_{N}}{R}-\frac{\delta v_{N}}{R}+\omega_{E}^{dr})tg\Phi_{E}\sin\Phi_{N}-(\frac{v_{E}}{R}tg\varphi +u\sin\varphi +\frac{\delta v_{E}}{R}tg\varphi +u\cos\varphi \delta \varphi +\\ +\frac{v_{E}}{R}\sec^{2}\varphi \delta \varphi +\omega_{up}^{dr})tg\Phi_{E}\cos\Phi_{N} \end{array}$$

where, $v_{N}{,\;v}_{E}{,\;v}_{up}$ denote velocity projection of the spacecraft along the axis of the geographic navigation frame; ${\delta v}_{N}{,\;\delta v}_{E}{,\;\delta v}_{up}$ denote projections of errors in determining the velocity of spacecraft along the axis of a geographic navigation frame; $\Phi_{N}{,\;\Phi }_{E}{,\;\Phi }_{up}$ denote deviation angles between platform and geographic navigation frame; $f_{N}{,\;f}_{E}{,\;f}_{up}$ denote projections of apparent acceleration of aircraft along the axis of geographic navigation frame; $\omega_{N}^{dr}{,\;\omega }_{E}^{dr}{,\;\omega }_{up}^{dr}$ denote projection of the drift of gyro-stabilized platform along the axis of geographic navigation frame; $\mu_{N}{,\;\mu }_{E}$ are coefficient errors of accelerometer; $B_{N}{,\;B}_{E}$ are zero offset of accelerometer; φ is local latitude; δφ is latitude error; *u* is the Earth rotation speed; *R* is the Earth radius.

When the INS is functioning over sufficiently long-time intervals, its errors could reach unacceptably large values. Therefore, it is necessary to compensate for the errors of an autonomous INS using the internal connections of the system

### 2.2. Method of Measurements Generation Based on Signals from Precession Angle Sensors

In the present compensation of autonomous INS errors, it is assumed that the generation of correction signals is proportional to the system errors in determining velocity and the angles of GSP deviations relative to the accompanying navigation frame and GSP drifts. In the absence of an external information sensor these autonomous INS errors cannot be directly measured and thus it is necessary to evaluate system errors using a filtering algorithm to generate a compensation signal. Similar to the equations of the object in filtering algorithm, equations of INS errors are expressed in terms of the angles of deviation of angles of GSP with respect to the reference coordinate system, and as measurements we can take the deviation angles of GSP from the horizontal plane and a given direction in the azimuth formed, on the basis of information received from sensors of the gyros precession angle.

Based on the descriptions above, the present method of measurements generation is described in more detail. Firstly, the movement of gyros with respect to the GSP could be described by the following system of equations:

(2) $$\{\begin{array}{c} J\ddot{\delta }+h\dot{\delta }+H{\dot{\Phi }}_{1}=H{\dot{\Phi }}_{2}\delta +H{\dot{\Phi }}_{3}\Phi_{2}+M_{1}\\ J\ddot{\lambda }+h\dot{\lambda }-H{\dot{\Phi }}_{2}=H{\dot{\Phi }}_{1}\lambda -H{\dot{\Phi }}_{3}\Phi_{1}+M_{2}\\ J\ddot{\vartheta }+h\dot{\vartheta }+H{\dot{\Phi }}_{3}=H{\dot{\Phi }}_{2}\vartheta -H{\dot{\Phi }}_{1}\Phi_{2}+M_{3} \end{array}$$

where $\Phi_{1}{,\Phi }_{2}{,\Phi }_{3}\;$are angular coordinates of the orientation of GSP relative to the selected accompanying navigation frame; δ, λ, ϑ are gyros precession angles; *J* denotes the second moment of inertia relative to the gyro precession axis; *H* denotes the inherent angular momentum of gyros; *h* is the specific moment of high-speed friction forces around precession axis of the gyroscope; and $M_{i,\;i=1,2,3}\;$represent other small disturbance moments with a random character, which will not be considered in this work.

Under the framework that other small perturbation moments are not included in the next modeling, equations of motion of gyros as a first approximation can be represented in the form as follows:

(3) $$\{\begin{array}{c} J\ddot{\delta }+h\dot{\delta }+H{\dot{\Phi }}_{1}=0\\ J\ddot{\lambda }+h\dot{\lambda }-H{\dot{\Phi }}_{2}=0\\ J\ddot{\vartheta }+h\dot{\vartheta }+H{\dot{\Phi }}_{3}=0 \end{array}$$

Considering that the precession angles are directly measured by the gyros angle sensors, the orientation angles of GSP in the first approximation can be defined in the following system:

(4) $$\{\begin{array}{l} \Phi_{1}=-\frac{1}{H}\int \left(J\ddot{\delta }+h\dot{\delta }\right)dt\\ \Phi_{2}=\frac{1}{H}\int \left(J\ddot{\lambda }+h\dot{\lambda }\right)dt\\ \Phi_{3}=-\frac{1}{H}\int \left(J\ddot{\vartheta }+h\dot{\vartheta }\right)dt \end{array}$$

With the orientation angles expression of GSP $\Phi_{1}{,\Phi }_{2}{,\Phi }_{3}\;$in (4), we substitute the expressions of first approximation into the initial system (2). Then, the angles of orientation of GSP in the second approximation could be determined as:

(5) $$\{\begin{array}{l} \Phi_{1}=-\frac{J}{H}\dot{\delta }-\frac{h}{H}\delta +\frac{1}{H}\int \left[(J\ddot{\lambda }+h\dot{\lambda }\right)\delta -\frac{1}{H}(J\ddot{\vartheta }+h\dot{\vartheta })(J\dot{\lambda }+h\lambda )]dt\\ \Phi_{2}=\frac{J}{H}\dot{\lambda }+\frac{h}{H}\lambda +\frac{1}{H}\int \left[(J\ddot{\delta }+h\dot{\delta }\right)\lambda +\frac{1}{H}(J\ddot{\vartheta }+h\dot{\vartheta })(J\dot{\delta }+h\delta )]dt\\ \Phi_{3}=-\frac{J}{H}\dot{\vartheta }-\frac{h}{H}\vartheta +\frac{1}{H}\int \left[(J\ddot{\lambda }+h\dot{\lambda }\right)\vartheta +\frac{1}{H}(J\ddot{\delta }+h\dot{\delta })(J\dot{\lambda }+h\lambda )]dt \end{array}$$

Consequently, after some manipulations the formal dependence in the orientation angles from the gyros precession angles of GSP is obtained. Furthermore, the orientation angles formed according to systems of Equations (4) and (5) can be used in the estimation algorithm as a measurement. However, it should be noted that the obtained angles $\Phi_{1}{,\Phi }_{2}{,\Phi }_{3}\;$from Equation (5) are somewhat different from the true GSP orientation angles. The existing difference is due to the fact that integrand functions are defined by successive approximation method. Thus, using the information from gyros angle sensors, it is possible to continuously calculate the orientation angles of GSP.

In the well-known compensation methods for INS errors such as autonomous and corrected methods from an external source of information using filtering algorithms, compensation for the azimuthal deviation of GSP relative to the accompanying navigation frame is not performed. It is considered here in order to explain the fact that the azimuthal position of the object is unobservable according to the measurements of position or velocity of the object. Therefore, the presented method of measurement generation for the filtering algorithm has a distinct advantage in terms of estimation of the azimuthal deviation angel of GSP, and its compensation in the output information of INS.

## 3. Correction of Autonomous INS by Kalman Filter

**Figure 1:**
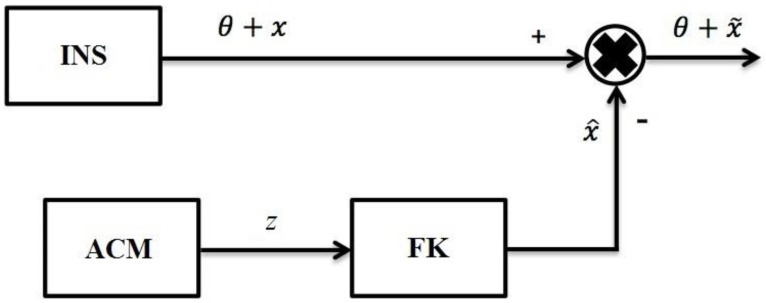
Scheme of autonomous INS correction by KF in the flight with constant velocity of aircraft. Where, θ—true information about the navigation parameters of a dynamic object; *x*—error vector of INS; $\tilde{x}$—estimation of error vector; $\hat{x}$—estimation of vector *x*; *z*—measurements with accelerometers; ACM—accelerometer; KF—Kalman filter.

It is known that the compensation of INS errors within a Kalman filter (KF) without the use of external measurements is for the case of aircraft motion with a constant velocity. Figure 1 shows a scheme for the correction of autonomous INS when the aircraft is flying at a constant velocity.

In the process of development of algorithmic correction for autonomous INS the known approaches have some disadvantages. The method of correction of autonomous INS involves only horizontal flight of the aircraft at a constant velocity. In this case, information from horizontal accelerometers is utilized as measurements. Let us consider the way of obtaining these measurements. First of all, GPS is assumed to deviate from the reference navigation frame at small angles as Φ_1_, Φ_2_, Φ_3_. In this case, it is possible to determine the projections of accelerations on the axis of GSP system:

(6) $${\left[\begin{array}{c} a_{x}\\ a_{y}\\ a_{z} \end{array}\right]}_{P}=\left[\begin{array}{ccc} 1 & \Phi_{3} & -\Phi_{2}\\ -\Phi_{3} & 1 & \Phi_{1}\\ \Phi_{2} & -\Phi_{1} & 1 \end{array}\right]{\left[\begin{array}{c} a_{x}\\ a_{y}\\ a_{z} \end{array}\right]}_{o}$$

where the index "*p* " denotes a GSP system and the index " o " denotes the reference navigation frame.

From Equation (6), we can further obtain the following expressions:

(7) $$\left\{ \begin{array}{l} a_{x}^{p}=a_{x}^{o}+\Phi_{3}a_{y}^{o}-\Phi_{2}a_{z}^{o}\\ a_{y}^{p}=-\Phi_{3}a_{x}^{o}+a_{y}^{o}+\Phi_{1}a_{z}^{o} \end{array}\right.$$

As we assumed above that the aircraft is moving at a constant velocity, the following relationship can be used: $a_{x}^{o}=a_{y}^{o}=0$. In addition, an assumption is made for the aircraft that during flight it has acceleration $a_{z}^{o}=g$. Then, substituting these relationships into Equation (7), equations can be written as follows:

(8) $$\left\{ \begin{array}{l} a_{x}^{p}=-\Phi_{2}g\\ a_{y}^{p}=\Phi_{1}g \end{array}\right.$$

However, in the real motion of an aircraft, the accelerometer readings suffer from individual calibration errors such as zero-offset and a scale factor error. Consequently, Equation (8) can be completely formulated as:

(9) $$\left\{ \begin{array}{l} a_{x}^{p}=-\Phi_{2}g+\delta a_{x}\\ a_{y}^{p}=-\Phi_{1}g+\delta a_{y} \end{array}\right.$$

where δ*a_x_*, δ*a_y_* are equivalent accelerometer errors, and then these signals from accelerometers can be used as measurements for the estimation algorithm:

(10) $$\left\{ \begin{array}{l} z_{1}=a_{x}^{p}=-\Phi_{2}g+\delta a_{x}\\ z_{2}=a_{y}^{p}=-\Phi_{1}g+\delta a_{y} \end{array}\right.$$

Subsequently, based on the whole scheme the errors of INS can be evaluated.

In practical applications, as a rule the aircraft performs a complex accelerated motion, considering of which the presented method is rarely used to compensate for autonomous INS errors. Then, a different approach using an estimation algorithm to improve the accuracy of autonomous INS should be proposed. In this model, the error equations of the autonomous INS are used as the objective equations in the estimation algorithm, and the angles obtained as signals from the precession angle sensors are taken as measurements. As an estimation algorithm, it is necessary to use an adaptive Kalman filter [11,12,13,14,15], capable of functioning in the absence of a priori information about the statistical characteristics of the inputs and measurement noises. It is necessary to apply the adaptive Kalman filter to the proposed method because in practice the covariance matrix of input noises including zero offset, accelerometer drift, and gyroscope drift is always unknown. Furthermore, due to the adopted approximations the a priori covariance matrix of the measuring noise, which includes the variances of errors in the generation of deviation angles of GPS, is also unknown.

According to the formulated measurements in Equation (5), the adaptive Kalman filter restores the entire state vector, including the INS errors in determining the velocity, deviation angles, and GSP drifts. Then, the estimate of the vector state is used to compensate for INS errors in the output information.

**Figure 2:**
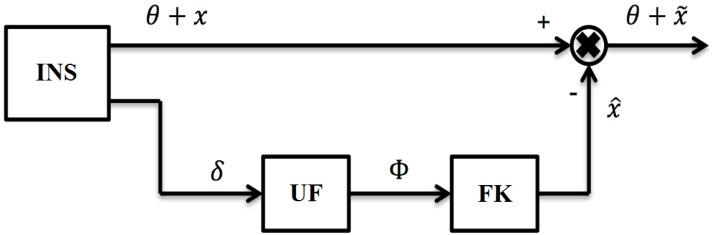
Scheme of autonomous INS correction using signals from precession angle sensors. Where δ denotes the signal from the precession angle sensor; UF represents unit of measurement generation; Φ indicates the formed measuring deviation angles of GSP.

This proposed method of INS error compensation allows sufficient compensation for system errors without using any external source of information. Namely, it helps to maintain the autonomy of the system. The autonomous INS correction scheme using signals from precession angle sensors is shown in Figure 2.

It is noted that the use of a scheme, as shown in Figure 2 allows to compensate for the errors of the autonomous INS in output signals, and the dynamics of the INS is preserved from the influence of such a compensation process.

## 4. Correction of Autonomous INS Using the Reduced Measurements

The formed orientation angles as described in the previous section have been verified to be used as measurements in the filtering algorithm, and the application of adaptive Kalman filter algorithms is carried out with the correction of highly accurate, but at the same time, expensive INS. Therefore, it is preferable to use simpler correction algorithms for serial INSs of the second accuracy class.

In the development of correction algorithms for aircraft motion, a nonlinear INS errors model is considered to generate measurement signals of directly unmeasured variables of the state vector. Firstly, the nonlinear model of INS errors should be simplified, and we will consider only the stationary part of the nonlinear equations since the specific motion parameters of the aircraft are not required to solve this part.

Based on the above description, it is assumed that angles Φ*_E_* and Φ*_N_* characterizing the errors of horizontal orientation remain small values throughout the work period of the INS and angle Φ*_up_*, which characterizes the errors of azimuth orientation, can increase indefinitely. Then, the non-linear error model of the INS is defined by the following system of equations:

(11) $$\{\begin{array}{l} \delta {\dot{v}}_{E}=-g\cos\Phi_{E}\sin\Phi_{N}+\sin B_{E}\\ \delta {\dot{v}}_{N}=g\sin\Phi_{E}+B_{N}\\ {\dot{\Phi }}_{E}=-\frac{\delta v_{N}}{R}\cos\Phi_{N}+\omega_{E}^{dr}\cos\Phi_{N}\\ {\dot{\Phi }}_{N}=\frac{\delta v_{E}}{R}-\frac{\delta v_{N}}{R}tg\Phi_{E}\sin\Phi_{N}+\omega_{N}^{dr} \end{array}$$

Additionally, the equations of INS errors can be written separately for each horizontal channel.

The error model of the Northern INS channel has the following form:

(12) $$\{\begin{array}{l} \delta {\dot{v}}_{N}=g\sin\Phi_{E}+B_{N}\\ {\dot{\Phi }}_{E}=-\frac{\delta v_{N}}{R}\cos\Phi_{N}+\omega_{E}^{dr}\cos\Phi_{N}\\ {\dot{\omega }}_{E}^{dr}=-\beta \omega_{E}^{dr}+A\sqrt{2\beta }\omega \end{array}$$

Moreover, here it is considered that the angle Φ*_E_* is small and thus we have the relationship that sinΦ*_E_* ≈ Φ*_E_*. Substituting them into Equation (12), then the system of equations can be rewritten in matrix form:

(13) $$x_{k}=Fx_{k-1}+w_{k-1}$$

where

$$x_{k}={\left[\begin{array}{c} \delta v_{N}\\ \Phi_{E}\\ \omega_{E}^{dr} \end{array}\right]}_{k};F=\left[\begin{array}{ccc} 1 & Tg & 0\\ -\frac{T}{R}\cos\Phi_{N} & 1 & T\cos\Phi_{N}\\ 0 & 0 & 1-T\beta \end{array}\right];\omega_{k}={\left[\begin{array}{c} TB_{N}\\ 0\\ TA\sqrt{2\beta }\omega \end{array}\right]}_{k}$$

Here, in Equation (13) w is white noise.

### 4.1. Method of Correction Signals Formation Using Non-Linear Errors Equations of INS

The method of forming the correction signals based on non-linear errors equations applicable for serial INSs with the second accuracy will be discussed. Each measurement step is divided into n sub-steps in the modeling process. These measurements are expressed through the state vector parameters of the first sub-step of the measurements. As the characteristics of the input, measurements and their noise are unknown, thus they could be neglected. At the same time, the state vector and the measurement equations can be expressed as follows:

(14) $$x_{k}=Fx_{k-1}$$

(15) $$z_{k}=Hx_{k}$$

Then, the equations for the time instants denoted with 2, 3, ..., n can be written down, where n is the dimension of vector ***x_k_***, and the vectors ***x*_2_**, ***x*_3_**, … by ***x*_1_** are formulated as follows [27]:

(16) $$\{\begin{array}{l} x_{2}=Fx_{1}\\ x_{3}=Fx_{2}=F^{2}x_{1}\\ \cdot \\ \cdot \\ \cdot \\ x_{n}=F^{n-1}x_{1} \end{array}$$

Accordingly, we can obtain the equations of measurements for the time instants 1, 2, ..., *n*:

(17) $$\{\begin{array}{l} z_{1}=Hx_{1}\\ z_{2}=Hx_{2}=HFx_{1}\\ z_{3}=Hx_{3}=HFx_{2}=HF^{2}x_{1}\\ \cdot \\ \cdot \\ z_{n}=Hx_{n}=HF^{n-1}x_{1} \end{array}$$

Additionally, the system of Equation (17) can be written in matrix form:

(18) $$Sx_{1}=z^{*}$$

where

$$S=\left[\begin{array}{c} H\\ HF\\ HF^{2}\\ \cdot \\ \cdot \\ HF^{n-1} \end{array}\right];z^{*}=\left[\begin{array}{c} z_{1}\\ z_{2}\\ z_{3}\\ \cdot \\ \cdot \\ z_{n} \end{array}\right]$$

From Equation (18), we can obtain the next expression:

(19) $$x_{1}=S^{-1}z^{*}$$

Here, $S^{-1}$ is the observability matrix of the system, which is nonsingular if the state vector is completely observable by measurements.

Hence, the correction signals for horizontal channels can be formed by using Equation (19).

### 4.2. Generation of Correction Signals for the Northern Channel

As it has been presented above that the equation for the object is formulated as:

(20) $$x_{k}=Fx_{k-1}$$

where the matrix of $x_{k}$ and **F** have the form, respectively,

$$x_{k}={\left[\begin{array}{c} \delta v_{N}\\ \Phi_{E}\\ \omega_{E}^{dr} \end{array}\right]}_{k};F=\left[\begin{array}{ccc} 1 & Tg & 0\\ -\frac{T}{R}\cos\Phi_{N} & 1 & T\cos\Phi_{N}\\ 0 & 0 & 1-T\beta \end{array}\right]$$

It is known that the equation of measurement has the form (15), in which the measurement matrix **H** can be defined as: H=[0 1 0]. In this case, the matrix **S** is determined by the following formula:

$$S=\left[\begin{array}{ccc} 0 & 1 & 0\\ -\frac{T\cos\Phi_{N}}{R} & 1 & T\cos\Phi_{N}\\ -\frac{2T\cos\Phi_{N}}{R} & 1-\frac{gT^{2}\cos\Phi_{N}}{R} & T(2-\beta T)\cos\Phi_{N} \end{array}\right]$$

and the inverse matrix ***S***^−1^ is defined by the formula:

$$S^{-1}=\left[\begin{array}{ccc} \frac{-gT^{2}\cos\Phi_{N}+R(\beta T-1)}{\beta T^{2}\cos\Phi_{N}} & \frac{R(2-T\beta )}{\beta T^{2}\cos\Phi_{N}} & -\frac{R}{\beta T^{2}\cos\Phi_{N}}\\ 1 & 0 & 0\\ -\frac{gT^{2}\cos\Phi_{N}+R}{R\beta T^{2}\cos\Phi_{N}} & \frac{2}{\beta T^{2}\cos\Phi_{N}} & -\frac{1}{\beta T^{2}\cos\Phi_{N}} \end{array}\right]$$

At the same time, using Equation (19), the correction signals for the Northern channel can be generated:

(21) $$\{\begin{array}{l} z(\delta v_{N})=\frac{-gT^{2}\cos\Phi_{N}+R(\beta T-1)}{\beta T^{2}\cos\Phi_{N}}z_{k}+\frac{R(2-T\beta )}{\beta T^{2}\cos\Phi_{N}}z_{k+1}-\frac{R}{\beta T^{2}\cos\Phi_{N}}z_{k+2}\\ z(\Phi_{E})=z_{k}\\ z(\omega_{E}^{dr})=-\frac{gT^{2}\cos\Phi_{N}+R}{R\beta T^{2}\cos\Phi_{N}}z_{k}+\frac{2}{\beta T^{2}\cos\Phi_{N}}z_{k+1}-\frac{1}{\beta T^{2}\cos\Phi_{N}}z_{k+2} \end{array}$$

In expression (21), the scalar measurements *z_k_*, *z_k_*_+1_, *z_k_*_+2_ represent functions of the precession angles. They are formed on the basis of information from the precession angle sensors of the corresponding gyroscope according to Equation (5). Furthermore, the signals from the precession angle sensors are averaged preliminarily and a vector of the reduced measurements is formed from the already smoothed signals.

Finally, a correction signal proportional to the vector of the given measurements is implemented for compensation of errors in the output information of INS. Thus, the proposed compensation provides the possibility of increasing the accuracy of an autonomous INS at the expense of internal links only.

## 5. Simulation Results of Autonomous INS Correction Algorithms Using Signals from Precession Angle Sensors

**Figure 3:**
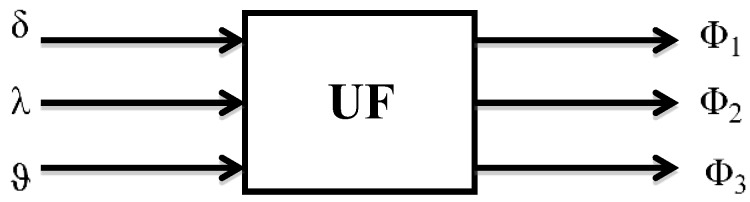
The scheme of signal generation for orientation angles of GSP. Where δ, λ, ϑ are precession angles of gyros, Φ_1_, Φ_2_, Φ_3_ are the orientation angles of GSP, and UF represents the unit of signal generation.

**Figure 4:**
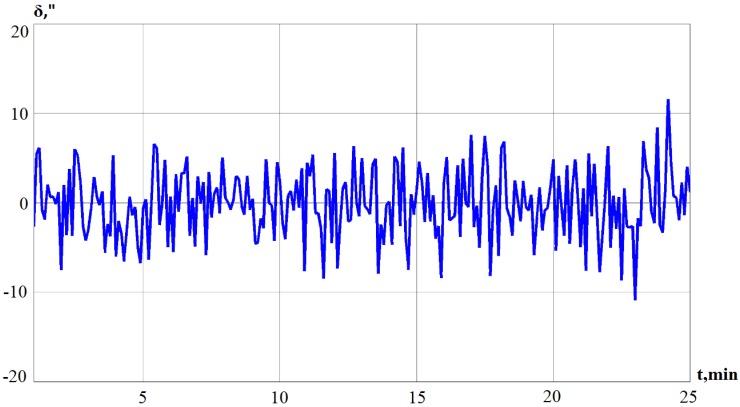
Signals received from the precession angle sensor.

To validate the proposed algorithms for correction of an autonomous INS, simulations with experimental data are executed under specific environment conditions. As described above, a scheme on how to constitute orientation angles is shown in Figure 3. The signals from the precession angle sensors of gyros, which are obtained during the laboratory experiment with the serial system of Ts060K (as shown in Figure 4), are forwarded as input to the unit of signal formation (UF), or they are formed in accordance with Equations (4) and (5). A laboratory experiment was conducted with a serial INS Ts060K. INS was installed on a three-stage test bed. During the experiment, vibrations were set on the test bed, and a situation when an aircraft performs harmonic and arbitrary oscillations around an axis in space is imitated. The projections of the external moment on the axis of test bed are as follows:

(22) $$\begin{array}{l} M_{x}=M_{x0}\sin\;\omega t\\ M_{y}=M_{y0}\sin\;\omega t\\ M_{z}=M_{z0}\sin\;\omega t \end{array}$$

where ω is the simulated oscillation frequency of the aircraft; *Mx*, *My*, *Mz*; *Mx*_0_, *My*_0_, *Mz*_0_ present the imitation of moments of external and inertial forces.

Signals of the GPS orientation angles are compiled in accordance with formulas (5). As shown in Figure 3, we can obtain information on the orientation angles of the GSP as signal output.

**Figure 5:**
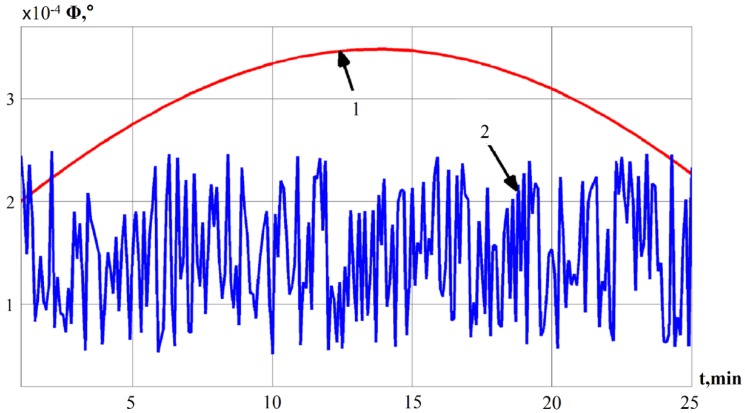
The deviation angle of GSP.

Using Equation (5), the deviation angle of GSP is obtained and the results are presented in Figure 5.

In Figure 5 there are two curves separately denoted as 1 and 2; line 1 indicates the deviation angle of the GSP, which is obtained by using a mathematical model of INS errors [28,29,30]; correspondingly, line 2 represents the deviation angle of GSP received from Equation (5).

In order to use the signal of the deviation angle of GSP, the signal needs to be smoothed in advance. Here, a smoothing method (moving average) is considered and implemented.

**Figure 6:**
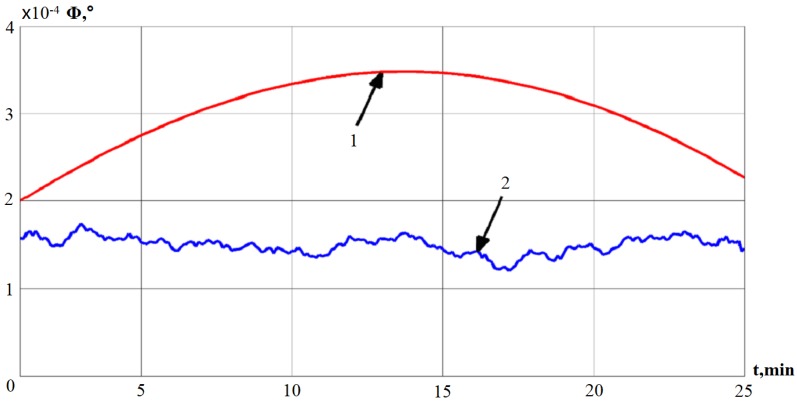
The deviation angle of GSP, obtained by the smoothing average method.

In Figure 6, line 1 represents the deviation angle of GSP obtained using the mathematical model, and line 2 represents the deviation angle of GSP, which is obtained by the smoothing average method.

As an estimation algorithm it is necessary to use an adaptive Kalman filter, capable to operate properly in the absence of a priori information on the statistical characteristics of the inputs and measurement noises. Therefore, a Kalman filter is considered to drive the simulations.

**Figure 7:**
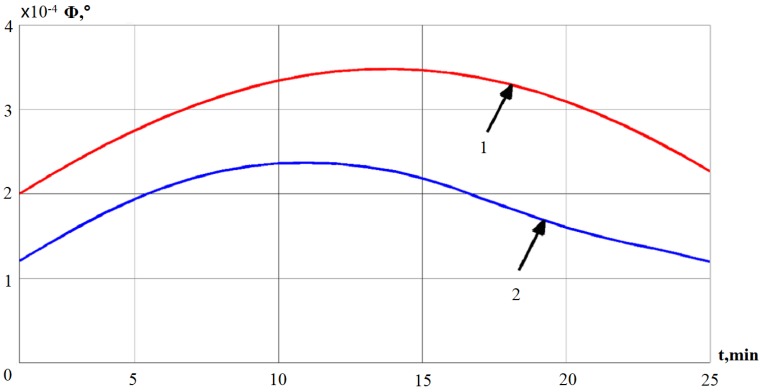
The deviation angle of GSP obtained by the Kalman filter.

Figure 7 illustrates the results of deviation angles obtained from the mathematical model with the help of the Kalman filter method. 1, 2 denote the mathematical model and Kalman filter separately.

**Figure 8:**
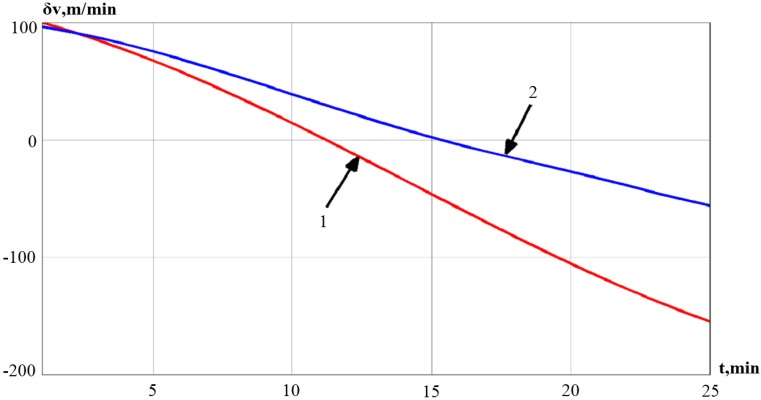
Error in determining the velocity and its estimation by the Kalman filter.

In Figure 8, line 1 represents the error in determining the velocity obtained by the mathematical model, and line 2 represents the error in determining the velocity obtained using the Kalman filter.

From the simulation results and comparison of modeling approaches, it is noted that we can obtain an estimate of the error in determining the velocity, the deviation angle of GSP, and the drift by the adaptive Kalman filter. The autonomous INS errors are compared with autonomous INS errors corrected in the output signal. After applying the adaptive Kalman filter for estimation of the algorithm, it can be seen that the accuracy of determining the velocity increases by an average of 44%. The deviation angle of GSP increases by 65% and the drift by 11%. 

Due to the fact that using the developed algorithm of measurement formation only a part of deviation angle of GPS is measured. The results of mathematical modeling of the GSP drift and its evaluation using the adaptive Kalman filter demonstrates low accuracy. The generated measurements are considered as inputs to the Kalman filter and they differ from the estimated process model, which is used in the Kalman filter.

## 6. Conclusions 

In this paper the autonomous navigation systems have been considered and various methods have been validated for their correction. Under the conditions of complex motion of an aircraft, nonlinear INS error models are always involved, where the use of nonlinear models for correction leads to an increase in accuracy consequently. It is advisable to use non-linear INS error models with a simple feature and it should be suitable for implementation in an on-board computer of aircraft to improve the accuracy of navigation definitions of autonomous INS. Considering that the INS refers to the second class of accuracy, in this work new algorithms for compensation of autonomous INS errors due to internal links based on signals from precession angle sensors have been developed, and an algorithm is presented for the correction of autonomous INS in the output signals based on the signals obtained from precession angle sensors. 

Furthermore, this paper proposes another algorithm for compensating directly immeasurable errors of an autonomous INS in the output signals using the adaptive Kalman filter, which formed the generated measurements for the Kalman filter using an INS error model and signals from precession angle sensors. Results of the mathematical modeling which demonstrated the efficiency and effectiveness of the developed algorithms are shown, also, the developed algorithmic methods can evidently improve the performance of the aircraft INS in an autonomous flight mode.
